# Temporal resistance variation of the second generation HTS tape during superconducting-to-normal state transition

**DOI:** 10.1186/2193-1801-2-599

**Published:** 2013-11-09

**Authors:** Vladimir A Malginov, Andrey V Malginov, Leonid S Fleishman

**Affiliations:** Lebedev Physical Institute RAS, Moscow, 119991 Russia; Krzhizhanovsky Power Engineering Institute, Moscow, 119991 Russia

**Keywords:** High-temperature superconductors, Quench, Normal zone propagation

## Abstract

**Background:**

The quench process in high-temperature superconducting (HTS) wires plays an important role in superconducting power devices, such as fault current limiters, magnets, cables, etc. The superconducting device should survive after the overheating due to quench.

**Methods:**

We studied the evolution of the resistance of the YBCO tape wire during the quench process with 1 ms time resolution for various excitation voltages.

**Findings:**

The resistive normal zone was found to be located in a domain of about 1-4 cm long. The normal state nucleation begins in 40-60 ms after voltage is applied across the HTS tape. In subsequent 200-300 ms other normal state regions appear. The normal domain heating continues in the following 5-10s that results in a factor of 2–3 increase of its resistance.

**Conclusions:**

Formation of the normal domain during the quench process follows the same stages for different excitation voltages. Characteristic domain sizes, lifetimes and temperatures are determined for all stages.

## Introduction

The quench process in high-temperature superconducting (HTS) wires plays an important role in superconducting fault current limiter operation. It occurs when current in a wire exceeds the critical value and as a result, the wire resistance becomes nonzero. The problem of quench stability is related to the heat transfer and is especially crucial for the Second Generation HTS wires on highly resistive substrates. We present here the results of studies of the normal zone generation.

## Methods and results

We studied the process of quench in HTS tapes using the experimental procedure described in (Fleishman et al., 2010). The sample was 12 mm wide and 100 mm long SuperPower YBCO tape SF12100 (Super-power). Both nominal and measured critical currents at 77 K are about 300A. It consists of 100 mu of Hastelloy substrate, 1 mu YBCO (critical temperature T_c_ = 91 K) and 1.5mu Ag layers. Measurements were performed with the tape immersed in liquid nitrogen. The AC (50 Hz) voltage step with the amplitude V_0_ was applied to the sample at the time t_0_. After that, during the subsequent 40s, we registered the current I and sample AC resistance Z with 1 ms time resolution.

**Figure 1:**
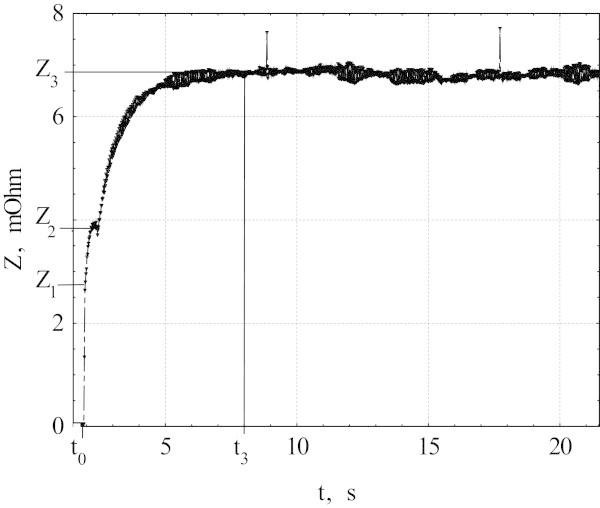
**The sample resistance versus time dependence.** AC voltage step of V_0_ = 379 mV was applied at the moment t_0_

**Figure 2:**
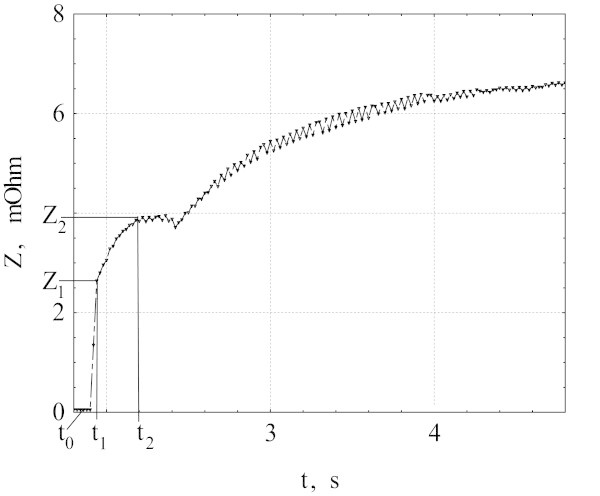
**The expanded view of the sample resistance vs. time dependence for the initial period (t < 5 s).** AC voltage step V_0_ = 379 mV was applied at the moment t_0_.

Figures 1 and 2 show the resistance Z as a function of time t for V_0_ = 379 mV. Time dependence of Z observed in all measurements may be divided into three stages. At the first stage the normal zone forms in a “weak” segment due to exceeding of the local critical current, and Z increases up to Z_1_ at the moment t_1_. At the second stage from t_1_ to t_2_, the normal region grows due to heat generation inside the initial normal zone, and Z increases up to Z_2_. At the third stage, t > t_2_, the resistance increases to the equilibrium value Z_3_ as a result of temperature growth in the newly formed normal domain and decrease of current.

**Figure 3:**
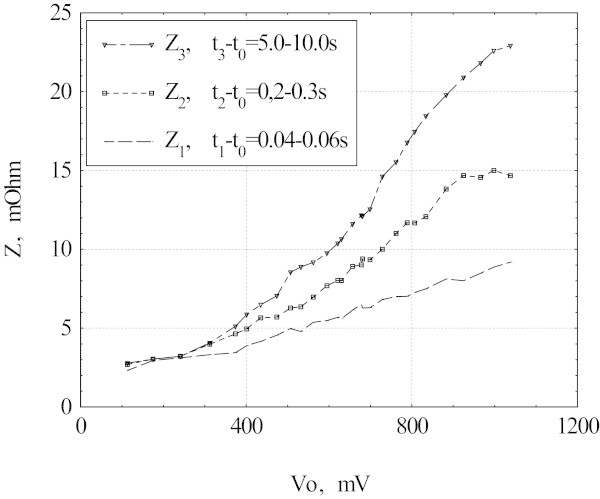
**Resistances Z**_**1**_ **, Z**_**2,**_ **and Z**_**3**_ **as the functions of the voltage V**_**0**_ **.**

The sample resistances Z_1_, Z_2,_ and Z_3_ as functions of voltage step magnitude V_0_ are shown in Figure 3. These resistances grow monotonically with V_0_. Up to V_0_ = 300 mV heating processes are weak and all the three stages merge. At V_0_ = 1 V the initial stage resistance Z_1_ is about 30% of the final value Z_3_.

The normal domain size can be estimated using the voltage dependence of the domain temperature and temperature dependence of the wire resistance. Maximal temperature T_M_(K) is expressed the following way (Mal’ginov et al., 2013):1

Equation (1) is experimentally proven to be valid in the range 0.5 V < V___0 < 0.8 V. In order to estimate the length of the normal zone we do assume that this formula is applicable also outside the specified range. The resistance Z (mOhm) of the zone where the YBCO layer is in the normal state (T(K) > T_c_) is given by the following expression:2

here L (mm) is the length of the zone where T > T_c_ for t > t_1_, V_0_ (mV) is the applied voltage magnitude.

Using (1) and (2) and assuming that Z_1_ is the domain resistance at liquid nitrogen temperature and Z_3_ is the resistance at the maximum temperature, one can obtain the domain size (L_1_) and the size of zone with T_M_ (L_3_) as function of V_0_:34

**Figure 4:**
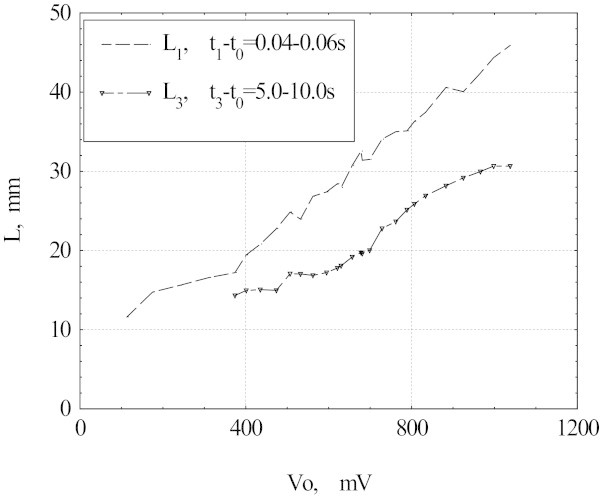
**The normal domain size (L**_**1**_ **) and the size of zone with maximum temperature (L**_**3**_ **) as a function of the voltage V**_**0**_ **.**

Figure 4 shows L_1_ and L_3_ values versus V_0_ calculated from (3) and (4).

## Conclusions

From the above results we conclude that during the superconducting-to-normal state transition in HTS tape the normal phase is limited to a single domain. The domain nucleates in 40-60 ms after the voltage is applied. In the subsequent 5-10s the domain heats up; it results in 2–3 times increase of the resistance. Central part of the domain is about 20-30 mm long. Inside the both of the 3-5 mm long edges of the domain the temperature falls from the maximal temperature T_M_ to 90 K.
